# (Acetato-κ*O*)(acetato-κ*O*,*O*′)bis­(1,3-diazinane-2-thione-κ*S*)cadmium(II)

**DOI:** 10.1107/S1600536812041852

**Published:** 2012-10-13

**Authors:** Rashid Mahmood, Saima Ghulam Hussain, Mohammed Fettouhi, Anvarhusein A. Isab, Saeed Ahmad

**Affiliations:** a Division of Science and Technology, University of Education, Township, Lahore, Pakistan; b Department of Chemistry, University of Engineering and Technology, Lahore 54890, Pakistan; c Department of Chemistry, King Fahd University of Petroleum and Minerals, Dhahran 31261, Saudi Arabia

## Abstract

In the title complex, [Cd(CH_3_COO)_2_(C_4_H_8_N_2_S)_2_], the Cd^II^ cation is coordinated by three acetate O atoms and two S atoms of Diaz [Diaz = 1,3-diazinane-2-thione = 3,4,5,6-tetra­hydro­pyrimidine-2(1*H*)-thione]. The Cd^II^ coordination is augmented by one considerably longer Cd—O bond of 2.782 (3) Å to a carboxyl­ate O atom. The resulting coordination polyhedron around the Cd^II^ cations can be described as a highly distorted octa­hedron. The Diaz ligand and the acetate anions are linked by N—H⋯O hydrogen-bonding inter­actions.

## Related literature

For crystal structures of Cd^II^ complexes of thio­nes, see: Ahmad *et al.* (2011 ▶, 2012 ▶); Altaf *et al.* (2011 ▶); Beheshti *et al.* (2007 ▶); Lobana *et al.* (2008 ▶); Nawaz *et al.* (2010) ▶; Moloto *et al.* (2003 ▶, 2007 ▶); Wang *et al.* (2002 ▶); Wazeer *et al.* (2007 ▶). For van der Waals radii, see: Bondi (1964 ▶). 
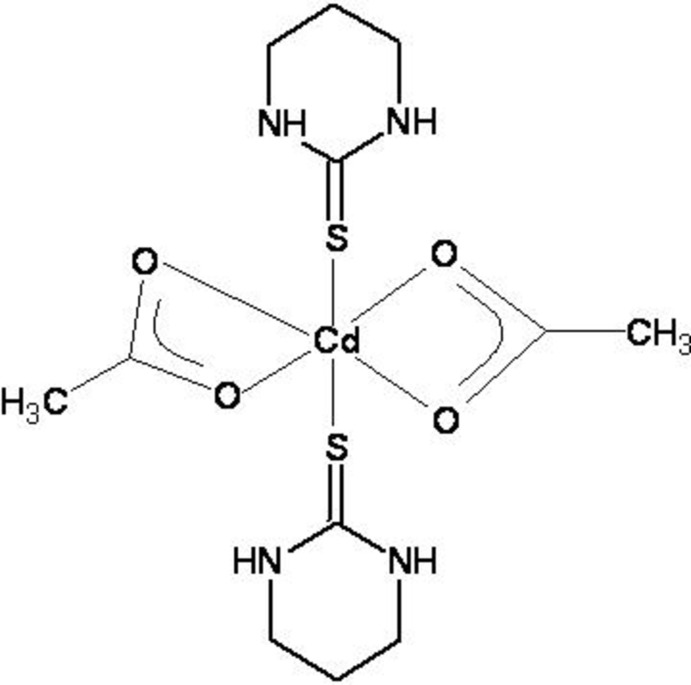


## Experimental

### 

#### Crystal data

[Cd(C_2_H_3_O_2_)_2_(C_4_H_8_N_2_S)_2_]
*M* *_r_* = 462.86
Triclinic, 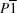

*a* = 8.6930 (17) Å
*b* = 10.175 (2) Å
*c* = 12.203 (2) Å
α = 97.452 (4)°
β = 100.683 (4)°
γ = 111.610 (3)°
*V* = 962.6 (3) Å^3^
*Z* = 2
Mo *K*α radiation
μ = 1.37 mm^−1^
*T* = 294 K
0.19 × 0.18 × 0.10 mm

#### Data collection

Bruker SMART APEX area-detector diffractometer
Absorption correction: multi-scan (*SADABS*; Sheldrick, 1996 ▶) *T*_min_ = 0.781, *T*_max_ = 0.875
13173 measured reflections
4775 independent reflections
3705 reflections with *I* > 2σ(*I*)
*R*_int_ = 0.024

#### Refinement

*R*[*F* ^2^ > 2σ(*F* ^2^)] = 0.033
*wR*(*F* ^2^) = 0.082
*S* = 1.02
4775 reflections
210 parameters
H-atom parameters constrained
Δρ_max_ = 0.47 e Å^−3^
Δρ_min_ = −0.43 e Å^−3^

### 

Data collection: *SMART* (Bruker, 2007 ▶); cell refinement: *SAINT* (Bruker, 2007 ▶); data reduction: *SAINT*; program(s) used to solve structure: *SHELXS97* (Sheldrick, 2008 ▶); program(s) used to refine structure: *SHELXL97* (Sheldrick, 2008 ▶); molecular graphics: *ORTEP-3* (Farrugia, 1997 ▶); software used to prepare material for publication: *publCIF* (Westrip, 2010 ▶).

## Supplementary Material

Crystal structure: contains datablock(s) I, global. DOI: 10.1107/S1600536812041852/nc2292sup1.cif

Structure factors: contains datablock(s) I. DOI: 10.1107/S1600536812041852/nc2292Isup2.hkl

Additional supplementary materials:  crystallographic information; 3D view; checkCIF report

## Figures and Tables

**Table 1:** Hydrogen-bond geometry (Å, °)

*D*—H⋯*A*	*D*—H	H⋯*A*	*D*⋯*A*	*D*—H⋯*A*
N1—H1⋯O3	0.86	1.94	2.779 (3)	167
N3—H3⋯O4	0.86	2.10	2.897 (4)	154
N2—H2⋯O2^i^	0.86	2.01	2.829 (3)	160
N4—H4⋯O1^ii^	0.86	2.03	2.836 (3)	156

Symmetry codes: (i) 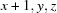; (ii) 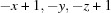.

**Fig. 1.**
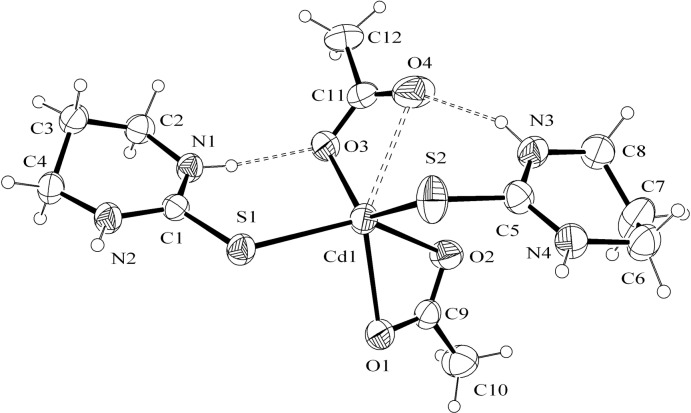
Molecular structure of the title compound showing the atomic numbering scheme. Displacement ellipsoids are drawn at the 30% probability level and dashed lines indicate intramolecular hydrogen bonding and the secondary Cd···O interaction.

## supplementary crystallographic information

### Comment

The structural reports on cadmium(II) complexes of thiones describe that in most of the cases, the cadmium(II) complexes exist as neutral monomeric (Ahmad *et al.* 2011, 2012; Beheshti *et al.*, 2007; Lobana *et al.* 2008; Nawaz *et al.*, 2010; Wazeer *et al.*, 2007) or polymeric (Moloto *et al.*, 2007; Wang *et al.*, 2002) with cadmium atom possessing a tetrahedral or distorted octahedral coordination environment respectively. In continuation of our studies on the structural chemistry of cadmium(II) complexes with thione ligands, we report here the crystal structure of the title compound. The crystal structure of the title complex (Figure 1) consists of discrete monomeric species in which the Cd(II) ion is bound to two sulfur Diaz atoms, two oxygen atoms belonging to a chelating acetate and a third oxygen atom of a second acetate anion. The Cd—S bond distances are 2.5787 (8) and 2.529 (1) Å respectively while the Cd—O bond distances are in the range of 2.266 (2)- 2.421 (2) Å. In addition, a secondary bonding interaction takes place with the second oxygen of one acetate anion with a distance of 2.782 (3) Å. The van der Waals radii for cadmium and oxygen are 1.58 and 1.52 Å respectively (Bondi, 1964). Considering the secondary bonding, the geometry could be considered as highly distorted octahedral. The S—Cd—S bond angle is 97.02 (3) °, the O—Cd—O bond angle for the bidentate acetate ligand is 53.86 (7) ° and the S—Cd—O bond angles vary from 92.37 (5) ° to 144.34 (6) °. The Cd—S and Cd—O bond lengths are in agreement with those reported for related compounds. Two intramolecular (N—H···O) hydrogen bonds are found between the Diaz ligands and acetate anions. The discrte complex molecules are also linked by intermolecular N—H···O hydrogen bonding (Table 1).

### Experimental

The title complex was prepared by adding 0.24 g (2.0 mmol) of 1,3-diazinane-2-thione in 15 ml of methanol to an aqueous solution (5 ml) of 0.26 g (1.0 mmol) cadmium sulfate in water followed by addition of 2 equivalents of sodium acetate in 10 ml water and stirring the mixture for 30 minutes. The colorless solution was filtered and the filtrate was kept at room temperature for crystallization. As a result, white crystalline product was obtained, that was washed with methanol and dried.

### Refinement

All H atoms were placed in calculated positions and were refined isotropic with *U*_iso_(H) = 1.2*U*_eq_(C,*N*) (1.5 for methyl H atoms) using a riding model with C—H distances of 0.96 Å and 0.97 Å and N—H distances of 0.86 Å.

### Crystal data

**Table:**

[Cd(C_2_H_3_O_2_)_2_(C_4_H_8_N_2_S)_2_]	*Z* = 2
*M**_r_* = 462.86	*F*(000) = 468
Triclinic, *P*1	*D*_x_ = 1.597 Mg m^−^^3^
Hall symbol: -P 1	Mo *K*α radiation, λ = 0.71073 Å
*a* = 8.6930 (17) Å	Cell parameters from 13173 reflections
*b* = 10.175 (2) Å	θ = 1.7–28.3°
*c* = 12.203 (2) Å	µ = 1.37 mm^−^^1^
α = 97.452 (4)°	*T* = 294 K
β = 100.683 (4)°	Block, colourless
γ = 111.610 (3)°	0.19 × 0.18 × 0.10 mm
*V* = 962.6 (3) Å^3^	

### Data collection

**Table:**

Bruker SMART APEX area-detector diffractometer	4775 independent reflections
Radiation source: normal-focus sealed tube	3705 reflections with *I* > 2σ(*I*)
Graphite monochromator	*R*_int_ = 0.024
ω scans	θ_max_ = 28.3°, θ_min_ = 1.7°
Absorption correction: multi-scan (*SADABS*; Sheldrick, 1996)	*h* = −11→11
*T*_min_ = 0.781, *T*_max_ = 0.875	*k* = −13→13
13173 measured reflections	*l* = −16→16

### Refinement

**Table:**

Refinement on *F*^2^	Primary atom site location: structure-invariant direct methods
Least-squares matrix: full	Secondary atom site location: difference Fourier map
*R*[*F*^2^ > 2σ(*F*^2^)] = 0.033	Hydrogen site location: inferred from neighbouring sites
*wR*(*F*^2^) = 0.082	H-atom parameters constrained
*S* = 1.02	*w* = 1/[σ^2^(*F*_o_^2^) + (0.0408*P*)^2^ + 0.0579*P*] where *P* = (*F*_o_^2^ + 2*F*_c_^2^)/3
4775 reflections	(Δ/σ)_max_ = 0.001
210 parameters	Δρ_max_ = 0.47 e Å^−^^3^
0 restraints	Δρ_min_ = −0.43 e Å^−^^3^

### Special details

**Table:**

Geometry. All e.s.d.'s (except the e.s.d. in the dihedral angle between two l.s. planes) are estimated using the full covariance matrix. The cell e.s.d.'s are taken into account individually in the estimation of e.s.d.'s in distances, angles and torsion angles; correlations between e.s.d.'s in cell parameters are only used when they are defined by crystal symmetry. An approximate (isotropic) treatment of cell e.s.d.'s is used for estimating e.s.d.'s involving l.s. planes.
Refinement. Refinement of *F*^2^ against ALL reflections. The weighted *R*-factor *wR* and goodness of fit *S* are based on *F*^2^, conventional *R*-factors *R* are based on *F*, with *F* set to zero for negative *F*^2^. The threshold expression of *F*^2^ > σ(*F*^2^) is used only for calculating *R*-factors(gt) *etc*. and is not relevant to the choice of reflections for refinement. *R*-factors based on *F*^2^ are statistically about twice as large as those based on *F*, and *R*- factors based on ALL data will be even larger.

### Fractional atomic coordinates and isotropic or equivalent isotropic displacement parameters (Å^2^)

**Table:**

	*x*	*y*	*z*	*U*_iso_*/*U*_eq_	
Cd1	0.71521 (3)	0.17566 (2)	0.769239 (17)	0.05732 (9)	
S1	0.98165 (9)	0.11886 (8)	0.77361 (6)	0.05721 (17)	
S2	0.73948 (12)	0.30728 (12)	0.60691 (8)	0.0827 (3)	
O1	0.5230 (3)	−0.0768 (2)	0.69890 (17)	0.0679 (5)	
O2	0.4199 (3)	0.0743 (2)	0.76067 (19)	0.0713 (6)	
O3	0.7698 (3)	0.2558 (2)	0.96021 (19)	0.0753 (6)	
O4	0.6916 (4)	0.4146 (3)	0.8923 (2)	0.1107 (10)	
N1	1.0593 (3)	0.1951 (2)	1.00174 (18)	0.0515 (5)	
H1	0.9615	0.2007	0.9917	0.062*	
N2	1.2447 (3)	0.1265 (3)	0.92374 (19)	0.0569 (6)	
H2	1.2731	0.0989	0.8638	0.068*	
N3	0.4916 (3)	0.3743 (3)	0.6630 (2)	0.0655 (6)	
H3	0.5640	0.4152	0.7283	0.079*	
N4	0.4398 (3)	0.2662 (3)	0.4764 (2)	0.0677 (7)	
H4	0.4757	0.2314	0.4233	0.081*	
C1	1.1027 (3)	0.1503 (3)	0.9105 (2)	0.0464 (5)	
C2	1.1658 (4)	0.2360 (3)	1.1187 (2)	0.0595 (7)	
H2A	1.1236	0.1581	1.1579	0.071*	
H2B	1.1597	0.3216	1.1596	0.071*	
C3	1.3496 (4)	0.2664 (3)	1.1175 (3)	0.0623 (7)	
H3A	1.4004	0.3573	1.0945	0.075*	
H3B	1.4150	0.2745	1.1935	0.075*	
C4	1.3539 (4)	0.1449 (3)	1.0350 (3)	0.0612 (7)	
H4A	1.4702	0.1670	1.0292	0.073*	
H4B	1.3143	0.0559	1.0624	0.073*	
C5	0.5411 (4)	0.3152 (3)	0.5810 (2)	0.0593 (7)	
C6	0.2707 (4)	0.2682 (4)	0.4463 (3)	0.0800 (10)	
H6A	0.1956	0.1855	0.3854	0.096*	
H6B	0.2784	0.3556	0.4196	0.096*	
C7	0.2012 (5)	0.2632 (5)	0.5474 (3)	0.0978 (13)	
H7A	0.1735	0.1679	0.5645	0.117*	
H7B	0.0962	0.2783	0.5305	0.117*	
C8	0.3232 (4)	0.3746 (4)	0.6498 (3)	0.0768 (9)	
H8A	0.3282	0.4695	0.6413	0.092*	
H8B	0.2835	0.3544	0.7174	0.092*	
C9	0.4005 (3)	−0.0511 (3)	0.7195 (2)	0.0561 (6)	
C10	0.2272 (4)	−0.1727 (4)	0.6978 (4)	0.0892 (11)	
H10A	0.2125	−0.2038	0.7676	0.134*	
H10B	0.2183	−0.2523	0.6416	0.134*	
H10C	0.1401	−0.1395	0.6704	0.134*	
C11	0.7493 (3)	0.3726 (3)	0.9725 (2)	0.0574 (7)	
C12	0.7991 (5)	0.4611 (3)	1.0914 (3)	0.0779 (10)	
H12A	0.9214	0.5038	1.1188	0.117*	
H12B	0.7504	0.4001	1.1406	0.117*	
H12C	0.7573	0.5363	1.0911	0.117*	

### Atomic displacement parameters (Å^2^)

**Table:**

	*U*^11^	*U*^22^	*U*^33^	*U*^12^	*U*^13^	*U*^23^
Cd1	0.05297 (13)	0.06353 (14)	0.05545 (14)	0.02626 (10)	0.00898 (9)	0.01124 (9)
S1	0.0544 (4)	0.0699 (4)	0.0480 (4)	0.0297 (3)	0.0097 (3)	0.0057 (3)
S2	0.0714 (5)	0.1273 (8)	0.0748 (6)	0.0542 (6)	0.0299 (4)	0.0473 (5)
O1	0.0557 (11)	0.0753 (13)	0.0716 (13)	0.0327 (10)	0.0119 (10)	−0.0007 (10)
O2	0.0577 (12)	0.0688 (13)	0.0832 (15)	0.0238 (10)	0.0231 (11)	−0.0012 (11)
O3	0.0630 (13)	0.0762 (14)	0.0839 (15)	0.0388 (11)	0.0061 (11)	−0.0071 (11)
O4	0.164 (3)	0.0721 (16)	0.0651 (16)	0.0344 (17)	−0.0127 (16)	0.0102 (12)
N1	0.0450 (11)	0.0592 (13)	0.0509 (13)	0.0223 (10)	0.0126 (9)	0.0096 (10)
N2	0.0503 (13)	0.0674 (14)	0.0554 (13)	0.0291 (11)	0.0123 (10)	0.0068 (11)
N3	0.0695 (16)	0.0747 (16)	0.0498 (14)	0.0336 (13)	0.0068 (11)	0.0033 (12)
N4	0.0780 (17)	0.0855 (18)	0.0496 (14)	0.0462 (15)	0.0164 (12)	0.0077 (12)
C1	0.0436 (13)	0.0442 (13)	0.0503 (14)	0.0163 (11)	0.0129 (10)	0.0093 (11)
C2	0.0680 (18)	0.0619 (17)	0.0464 (15)	0.0261 (14)	0.0098 (13)	0.0115 (12)
C3	0.0562 (17)	0.0582 (16)	0.0622 (18)	0.0188 (13)	0.0008 (13)	0.0114 (13)
C4	0.0504 (15)	0.0641 (17)	0.0674 (18)	0.0255 (14)	0.0046 (13)	0.0162 (14)
C5	0.0674 (18)	0.0646 (17)	0.0522 (16)	0.0305 (15)	0.0159 (14)	0.0206 (13)
C6	0.078 (2)	0.100 (3)	0.0587 (19)	0.045 (2)	0.0041 (16)	−0.0017 (17)
C7	0.071 (2)	0.147 (4)	0.068 (2)	0.046 (2)	0.0096 (18)	0.000 (2)
C8	0.078 (2)	0.093 (2)	0.066 (2)	0.0462 (19)	0.0191 (16)	0.0012 (17)
C9	0.0519 (15)	0.0685 (18)	0.0475 (15)	0.0267 (14)	0.0096 (12)	0.0082 (13)
C10	0.066 (2)	0.077 (2)	0.110 (3)	0.0162 (18)	0.0223 (19)	0.004 (2)
C11	0.0501 (15)	0.0525 (16)	0.0573 (17)	0.0102 (12)	0.0139 (12)	0.0025 (13)
C12	0.093 (2)	0.0612 (19)	0.0603 (19)	0.0145 (17)	0.0210 (17)	−0.0023 (15)

### Geometric parameters (Å, º)

**Table:**

Cd1—O3	2.266 (2)	C2—C3	1.515 (4)
Cd1—O2	2.364 (2)	C2—H2A	0.9700
Cd1—O1	2.421 (2)	C2—H2B	0.9700
Cd1—S2	2.5291 (10)	C3—C4	1.507 (4)
Cd1—S1	2.5787 (8)	C3—H3A	0.9700
Cd1—O4	2.782 (3)	C3—H3B	0.9700
S1—C1	1.721 (3)	C4—H4A	0.9700
S2—C5	1.728 (3)	C4—H4B	0.9700
O1—C9	1.245 (3)	C6—C7	1.470 (5)
O2—C9	1.247 (3)	C6—H6A	0.9700
O3—C11	1.259 (4)	C6—H6B	0.9700
O4—C11	1.215 (4)	C7—C8	1.492 (5)
N1—C1	1.319 (3)	C7—H7A	0.9700
N1—C2	1.462 (3)	C7—H7B	0.9700
N1—H1	0.8600	C8—H8A	0.9700
N2—C1	1.329 (3)	C8—H8B	0.9700
N2—C4	1.451 (3)	C9—C10	1.505 (4)
N2—H2	0.8600	C10—H10A	0.9600
N3—C5	1.320 (4)	C10—H10B	0.9600
N3—C8	1.444 (4)	C10—H10C	0.9600
N3—H3	0.8600	C11—C12	1.499 (4)
N4—C5	1.322 (4)	C12—H12A	0.9600
N4—C6	1.456 (4)	C12—H12B	0.9600
N4—H4	0.8600	C12—H12C	0.9600
			
O3—Cd1—O2	89.05 (8)	H3A—C3—H3B	108.3
O3—Cd1—O1	114.48 (8)	N2—C4—C3	109.3 (2)
O2—Cd1—O1	53.86 (7)	N2—C4—H4A	109.8
O3—Cd1—S2	132.00 (7)	C3—C4—H4A	109.8
O2—Cd1—S2	105.07 (6)	N2—C4—H4B	109.8
O1—Cd1—S2	110.65 (6)	C3—C4—H4B	109.8
O3—Cd1—S1	96.66 (5)	H4A—C4—H4B	108.3
O2—Cd1—S1	144.34 (6)	N3—C5—N4	119.3 (3)
O1—Cd1—S1	92.37 (5)	N3—C5—S2	121.1 (2)
S2—Cd1—S1	97.02 (3)	N4—C5—S2	119.5 (2)
O3—Cd1—O4	49.63 (8)	N4—C6—C7	109.1 (3)
O2—Cd1—O4	81.01 (8)	N4—C6—H6A	109.9
O1—Cd1—O4	134.07 (8)	C7—C6—H6A	109.9
S2—Cd1—O4	86.90 (6)	N4—C6—H6B	109.9
S1—Cd1—O4	128.48 (7)	C7—C6—H6B	109.9
C1—S1—Cd1	112.35 (9)	H6A—C6—H6B	108.3
C5—S2—Cd1	98.79 (10)	C6—C7—C8	112.5 (3)
C9—O1—Cd1	91.30 (17)	C6—C7—H7A	109.1
C9—O2—Cd1	93.95 (17)	C8—C7—H7A	109.1
C11—O3—Cd1	105.62 (19)	C6—C7—H7B	109.1
C11—O4—Cd1	81.81 (19)	C8—C7—H7B	109.1
C1—N1—C2	124.6 (2)	H7A—C7—H7B	107.8
C1—N1—H1	117.7	N3—C8—C7	110.3 (3)
C2—N1—H1	117.7	N3—C8—H8A	109.6
C1—N2—C4	122.8 (2)	C7—C8—H8A	109.6
C1—N2—H2	118.6	N3—C8—H8B	109.6
C4—N2—H2	118.6	C7—C8—H8B	109.6
C5—N3—C8	124.0 (2)	H8A—C8—H8B	108.1
C5—N3—H3	118.0	O1—C9—O2	120.9 (3)
C8—N3—H3	118.0	O1—C9—C10	120.2 (3)
C5—N4—C6	123.5 (2)	O2—C9—C10	118.9 (3)
C5—N4—H4	118.3	C9—C10—H10A	109.5
C6—N4—H4	118.3	C9—C10—H10B	109.5
N1—C1—N2	119.2 (2)	H10A—C10—H10B	109.5
N1—C1—S1	122.82 (19)	C9—C10—H10C	109.5
N2—C1—S1	117.95 (19)	H10A—C10—H10C	109.5
N1—C2—C3	110.1 (2)	H10B—C10—H10C	109.5
N1—C2—H2A	109.6	O4—C11—O3	122.5 (3)
C3—C2—H2A	109.6	O4—C11—C12	119.9 (3)
N1—C2—H2B	109.6	O3—C11—C12	117.6 (3)
C3—C2—H2B	109.6	C11—C12—H12A	109.5
H2A—C2—H2B	108.2	C11—C12—H12B	109.5
C4—C3—C2	109.2 (2)	H12A—C12—H12B	109.5
C4—C3—H3A	109.8	C11—C12—H12C	109.5
C2—C3—H3A	109.8	H12A—C12—H12C	109.5
C4—C3—H3B	109.8	H12B—C12—H12C	109.5
C2—C3—H3B	109.8		

### Hydrogen-bond geometry (Å, º)

**Table:**

*D*—H···*A*	*D*—H	H···*A*	*D*···*A*	*D*—H···*A*
N1—H1···O3	0.86	1.94	2.779 (3)	167
N3—H3···O4	0.86	2.10	2.897 (4)	154
N2—H2···O2^i^	0.86	2.01	2.829 (3)	160
N4—H4···O1^ii^	0.86	2.03	2.836 (3)	156

Symmetry codes: (i) *x*+1, *y*, *z*; (ii) −*x*+1, −*y*, −*z*+1.
